# An updated review of fish species reintroductions: global lessons to inform future riverine fish conservation in the UK

**DOI:** 10.1007/s44353-025-00072-w

**Published:** 2026-01-09

**Authors:** Reagan H. Pearce, Carl D. Sayer, Michael A. Chadwick

**Affiliations:** 1 Department of Geography, University College London, (North West Wing), Gower Street, London, WC1E 6BT UK; 2 Department of Geography, King’s College London, 40 Bush House (North East Wing, Aldwych), London, WC2B 4BG UK

**Keywords:** Freshwater fishes, Species reintroductions, Conservation, Translocations, Burbot

## Abstract

Reintroductions are potentially an effective conservation tool in freshwater conservation, including for fishes, where 37% are threatened in Europe alone (Tatár et al. (Tatár et al. in Oryx 514:718–729, 2017)). This review examines general and fish-specific species reintroductions to assess fish species reintroductions as a conservation tool, with a case study focus on the currently extirpated burbot *Lota lota* in the United Kingdom. Globally, there have been 324 published fish reintroductions since 1989, with a spatial bias to North America (*n* = 223). Common pitfalls identified for fish species reintroductions are not addressing the initial causes of decline, poor quality release habitat, and issues surrounding stocking, in relation to source population genetics. When considering the potential reintroduction of the burbot to the UK, many challenges remain including those that have been encountered in past projects for other fishes. For example, meeting policy requirements, designing and implementing a stocking strategy, and developing a comprehensive post-monitoring strategy are crucial to all future fish reintroduction attempts. Overall, a concerted effort is needed to disseminate species reintroduction project findings so that lessons, including recommendations and common pitfalls, can benefit future local conservation efforts.

## Introduction

Freshwater environments are some of the most threatened ecosystems globally [38, 136]. The most recent Living Planet Report by WWF found that between 1970 and 2020 freshwater wildlife populations have declined by 85% globally [155]. Comparable trends have also been observed in Europe, with some 50% of freshwater species having threatened status [52, 132]. Species reintroductions are increasingly applied as conservation tools, though fish reintroduction attempts remain less common compared to other taxa [13].

According to the International Union for the Conservation of Nature (IUCN), species translocations, which are simply the movement of a species from one area to another [72], have ecological conservation as the primary objective [70]. The term species reintroduction, while still a form of translocation, usually refers to the return of an (locally or nationally) extirpated species to a previously occupied area [2]. Historically, there have been many reasons for species reintroductions: hunting; pest control; aesthetic value; wildlife rehabilitation; and for conservation objectives [21]. Jachowski et al. [72] argue that species reintroductions push beyond the traditional boundaries of conservation, which is holding the line against negative anthropogenic impacts, to return lost species and restore the ecological conditions and complexities of past ecosystems. It is hard to define the success and failure of a reintroduction programme as their planning, methods, monitoring, and budget all vary. The international framework set out by the IUCN is highly useful, however, as it has helped to standardise approaches to species reintroductions [70]. The most crucial aspects to species reintroductions highlighted by the IUCN are: deciding when translocation is appropriate; release strategy including planning, site selection, and assessment; designing the post-release monitoring programme; feasibility and design to incorporate social and biological factors; risk assessments; and dissemination of information [70]. The IUCN’s Conservation Translocation Specialist Group state that over 1,500 species have been translocated globally [26].

Despite the acknowledged difficulties in planning and executing, species reintroductions have the potential to protect and improve biodiversity, reverse extinctions, and restore ecosystem functioning [55], which is crucial in currently deteriorating freshwater environments. Nevertheless, species reintroduction attempts (general and fish-specific reintroductions) have suffered from a series of pitfalls hindering their effectiveness or even resulting in failure. To utilise species reintroductions effectively for the benefit of freshwater ecosystems and biodiversity, it is important to understand common causes of failure and adapt new projects accordingly to maximise the chance of success. This review aims to assess fish species reintroductions as a conservation tool, with a specific focus on the United Kingdom (UK), by (i) gleaning lessons learnt from species reintroduction history, (ii) reviewing cases of fish reintroductions globally, and (iii) utilising past lessons in species reintroduction history to identify future considerations for a potential fish reintroduction project in the UK by using a case study of the burbot, *Lota lota*. While the review is for the benefit of fish reintroductions, it is informed by and has relevance to all species be they aquatic or terrestrial.

## A brief history of species reintroductions

While species reintroductions for conservation purposes have accelerated in recent years, they have a long history. Australasia has been a particular hotspot, having the first recorded translocations for conservation purposes [116]. In the 1880s, large numbers of kakapo *Strigops habroptilus* and kiwi *Apteryx australis*, were moved to an offshore island marking the first attempt to protect New Zealand’s native species from the impacts of exotic mammalian predators [116]. Seddon and Armstrong [115] record a detailed history of species reintroductions, with releases of species aimed at restoring wild populations being recorded as early as the 1910s. Despite the practice’s early origins, it was not until the 1960s and 70s that some successful reintroduction attempts began to raise the profile of this approach as a conservation tool. For example, in the United States (US), the peregrine falcon *Falco peregrinus* was subject to a captive breeding and release programme in the 1970s and in combination with the ban of DDT (an organochlorine pesticide), raised breeding pairs from ~ 40 in 1975 to over 1,600 pairs by 1999 [61]. Another reintroduction motivated by conservation objectives was the Arabian oryx *Oryx leucoryx* in the Middle East, where the wild population was rendered extinct by the mid-1970s, except for a few individuals caught in Oman by Phoenix Zoo, Arizona, US. The capture and later reintroduction of a few individuals at Jiddat al-Harasis, Oman in 1982 [102] was successful in increasing the wild population to 1,100 individuals with 6,000–7,000 estimated to be in captivity as of 2017 [1, 71].

Species reintroductions have had mixed results, and a lack of documentation of pitfalls, has not benefitted subsequent reintroduction programmes. It was not until the 1970s that legal and scientific frameworks for reintroductions began to be implemented, with 1977 seeing conferences organised to discuss correct handling procedures [126]. Eventually, in 1987, the IUCN gave a statement on species translocations [68]. Within a year, the IUCN’s specialist group on species reintroductions had established itself [103], but it was not until 1995 that the IUCN’s guidelines on species reintroductions became official policy [69], with these updated again in 2013 [70]. Research and collaboration within the scientific community on species reintroductions have become more common in recent decades.

It is clear from existing studies that species reintroductions can bring holistic benefits to an ecosystem and society. For example, species that may have socio-economic value (e.g., aesthetic, recreational, cultural, see [73]) can be popular with the public and have the benefit of increasing public engagement with conservation work, affording potential opportunities for funding [21]. Moreover, when species are reintroduced, their legal status can give them protections that extend to other species. For example, the piping plover *Charadrius melodus* is protected under the US Endangered Species Act and as a beach-nesting bird, its legal protection extend benefits to the flora and fauna of the barrier beach ecosystem it inhabits [60]. Also, prior to any reintroduction, a programme may require the restoration of habitat to a better quality in preparation for the new species, which improves habitat integrity and quality, thus being potentially beneficial to other species [101]. Depending on the species reintroduced, whether a keystone species or ecosystem engineer, there can also be direct ecological impacts from its reintroduction. For example, the reintroduction of top-level predator grey wolf *Canis lupus lupus* to Yellowstone national park, US is a widely cited example of ecological reintroduction success due to the restoration of trophic interactions between wolf, elk *Cervus elaphus* and willow *Salix* spp., which allowed for ecosystem restructuring across multiple trophic levels [119].

Species reintroductions, however, have limitations. They are often expensive, require extensive planning, and are complex, as each species has its own requirements for survival, hence standardisation is a challenge [21]. Nevertheless, common pitfalls have emerged, which can help to improve the chances of success of future projects if better disseminated. During an analysis of the IUCN’s *Global Reintroduction Perspective Series*, Berger‐Tal et al. [13] analysed 293 species translocation studies for common difficulties faced by project implementers. The study identified over 1,200 individual difficulties, reflecting how each species reintroduction faces its own unique challenges as a product of individual species requirements, project design, and available resources. It was concluded that the key pillars of reintroduction success are having good quality release habitat, understanding the species' biology and behaviour, and having an efficient post-release monitoring programme. While the most common pitfalls were evidenced, the severity of each pitfall and its resulting impact was not investigated [13]. From all the reintroductions observed in Berger-Tal et al. [13], a taxonomic bias towards birds and mammals was noted and of 293 animal translocations, 20 were amphibians, 28 invertebrates, 35 fish, 37 reptiles, 66 birds, and 106 were mammal translocations. Similarly, in their study of 699 reintroductions from IUCN data, Seddon et al. [116] found vertebrates to be overrepresented compared to their prevalence in nature. Specifically, mammals and birds were overrepresented, and fish were underrepresented. Interestingly, over half of the birds being reintroduced were classified as ‘Least Concern’ by the IUCN, suggesting that species reintroductions are driven more by national than international agendas and priorities [116].

Another concern presented by species reintroductions is disease risk. This not only relates to reintroduced species introducing or spreading new diseases in the environment, but also to pathogens or parasites that may prevent a species from surviving in its new environment [21]. Overall, conservation-based species translocations are regarded as a minimal threat in the spread of disease compared to invasive species, but it is still important to contemplate this risk when planning species reintroductions [70].

Other challenges that species reintroductions must overcome are public attitudes towards the candidate species. In the Vosges Massif, France, the reintroduction of Eurasian lynx *Lynx lynx* was undertaken over 1983—1993 [135]. Immediately after some releases, lynx were illegally killed, which was not anticipated by the reintroduction organisers as they had not undertaken sufficient research into public attitudes toward the species [146]. Ultimately, the reintroduced population failed to become self-sustaining due to human-induced mortality factors, especially including illegal killing [135]. Commonly, the main pitfalls that large predator reintroductions must overcome are perceived worries about public safety and conflict with agriculture as farmers often fear predation will impact on livestock [40, 144, 145].

An increasing challenge in managing species reintroductions is climate change scenarios [98]. Carter et al. [21] argue that, in combination with extensive habitat fragmentation and anthropogenic pressures, an increasing number of species will require human assistance under future climate change. This need is reflected in the development of a new sub-section in the IUCN guidelines [70], including ‘assisted colonisation’ and ‘ecological replacements'. The former is a new type of translocation where species will have to be moved to ensure their long-term survival and the latter involves identifying and translocating replacement species for those that are likely to be lost under future environmental change [115].

It is clear that species reintroductions generally have potential to bring direct ecological and indirect socio-economic benefits when applied as conservation tools, but they suffer from common pitfalls. Indeed, when considering freshwater fish reintroductions, specifically, a similar trend emerges.

## Fish reintroductions – Global overview

Freshwater fishes have higher extinction rates than many terrestrial taxa [107], likely due to freshwater habitats persistent stresses by anthropogenic pressures and climate change [24]. Common freshwater fish conservation strategies are habitat restoration, removal of invasive species, and stocking [24], but reintroductions are becoming an increasingly popular tool. Despite an underrepresentation in reintroduction programmes on a global scale, fish reintroductions have been extensive enough to allow for a review of best practice and of pitfalls. From published studies, Cochran-Biederman et al. [24] identified 260 individual fish reintroductions, distinguished by species, location, or method, from 1989 to 2013 from 75 published studies. Assessing these cases on 23 author-defined and 3 biological variables, covariates of success and failure could be determined. Table 1 summarises a further 64 fish reintroductions, including some previously unidentified by Cochran-Biederman et al. [24], and additional cases published from 2014 to 2024.

**Table 1:** Additional fish translocations unmentioned by Cochran‐Biederman et al. [24] in addition to cases published from 2014 to 2024

Scientific name	Common name	Location	Year	Source
		North America		
*Oncorhynchus tshawytscha*	Chinook salmon	McKenzie River, USA	Since 1996	Banks et al. [5]
		Lookingglass Creek, USA	Since 2001	Nuetzel et al. [90]
*Empetrichthys latos*	Pahrump poolfish	Springs Preserve, USA	2018	Saumure et al. [111]
*Moapa coriacea*	Moapa dace	Muddy River, USA	Since 2008	Syzdek et al. [124]
*Gasterosteus aculeatus*	Three-spined stickleback	Mountain Lake, USA		Young [156]
*Cottus cognatus*	Slimy sculpin	Driftless Region streams, USA	Since 2003	Huff [67]
*Oncorhynchus mykiss aquilarum*	Eagle Lake rainbow trout	Eagle Lake, USA	Long-term	Carmona-Catot and Moyle [19]
*Oncorhynchus clarkii lewisi*	Westslope cutthroat trout	Cherry Creek, USA	Since 1997	Kruse et al. [77]
*Oncorhynchus virginalis*	Rio Grande cutthroat trout	Rio Grande River, USA	Since 1998	Kruse et al. [77]
*Noturus baileyi*	Smoky madtom	Little Tennessee River, USA	Since 1986	Shute et al. [117]
*Noturus flavipinnis*	Yellowfin madtom	Upper Tennessee River, USA	Since 2003	Shute et al. [117]
*Salvelinus confluentus*	Bull trout	Yakima River, USA	Since 2018	Hayes and Banish [59]
		Glacial Lake National Park, USA	Since 2014	Downs and Fredenberg [37]
		Clackamas Basin, USA	Since 2013	Barrows et al. [6]
		Wallowa River, USA	1997	Whitesel et al. [141]
*Fundulus sciadicus*	Plains topminnow	Great Plains, USA	2014–2016	Schumann et al. [113]
*Atractosteus spatula*	Alligator gar	Western Kentucky, USA	2009–2014	Richardson [108]
*Scaphirhynchus platorynchus*	Shovelnose sturgeon	Bighorn River, USA	1996–2020	Hogberg et al. [65]
*Xyrauchen texanus*	Razorback sucker	San Juan River, USA	Since 2000	Diver et al. [35]
*Meda fulgida*	Spikedace	Blue River, USA	2012–2016	Hickerson et al. [63]
*Rhinichthys cobitis*	Loach minnow	Blue River, USA	2012–2016	Hickerson et al. [63]
*Gila robusta*	Roundtail chub	Blue River, USA	2012–2016	Hickerson et al. [63]
*Oncorhynchus nerka*	Okanagan sockeye salmon	Skaha Lake, BC, Canada	Since 2015	Blanchet et al. [14]
*Coregonus hoyi*	Bloater	Lake Ontario, Canada	2012–2020	Weidel et al. [138]
*Salmo salar*	Atlantic salmon	Inner Bay of Fundy, Canada	Since 2015	Bryson et al. [17]
*Coregonus huntsman*	Atlantic whitefish	Anderson Lake, Canada	2005–2012	Bradford et al. [16]
*Moxostoma hubbsi*	Copper redhorse	Rivière Richelieu, Canada	2004–2018	Lamothe et al. [78]
*Acipenser transmontanus*	White sturgeon	Upper Fraser River, Canada	Since 1990	Lamothe et al. [78]
*Zoogoneticus tequila*	Tequila splitfin	Teuchitlán River, Mexico	Since 2012	Domínguez et al. [36]
*Notropis boucardi*	Morelos minnow	Barranca de Chapultepec streams, Mexico		Contreras-MacBeath et al. [27]
		*Oceania*		
*Galaxias fuscus*	Barred galaxias	Victoria, Australia	2010	Ayres et al. [3]
*Galaxias pedderenis*	Pedder galaxias	Tasmania, Australia	1991–2007	Chilcott et al. [23]
*Paragalaxias mesotes*	Arthurs paragalaxias	Tasmania, Australia	2004–2013	Lintermans et al. [81]
*Galaxias auratus*	Golden galaxias	Tasmania, Australia	1996–1998	Hardie [57]
*Gadopsis bispinosus*	Two-spined blackfish	Australian Capital territory, Australia	2004–2006	Lintermans et al. [81]
*Gadopsis marmoratus*	River blackfish	South Australia	2011	Hammer et al. [56]
*Macquaria australsica*	Macquarie perch	New South Wales, Australia	1987–2014	Lintermans et al. [81]
		Australian Capital territory, Australia	Since 2006	Lintermans et al. [81]
		Victoria, Australia	Since 1990	Lintermans et al. [81]
*Tandanus tandanus*	Freshwater catfish	Victoria, Australia	1998–2008	Lintermans et al. [81]
*Maccullochella ikei*	Eastern freshwater cod	New South Wales, Australia	1989–2003	Nock et al. [88]
*Maccullochella mariensis*	Mary River cod	Queensland, Australia	1983–2011	Lintermans et al. [81]
*Ambassis agassizi*	Olive perchlet	New South Wales, Australia	2010–2014	Lintermans et al. [81]
*Melanotaenia eachamensis*	Lake Eacham rainbowfish	Queensland, Australia	1989	Lintermans et al. [81]
*Melanotaenia sp.*	Running River rainbowfish	Running River, Australia	Since 2015	Moy et al. [86]
*Melanotaenia sp.*	Malanda rainbowfish	North Johnstone River, Australia	2016	Moy et al. [85]
*Nannoperca obscura*	Yarra pygmy perch	Murray-Darling Basin, Australia	2011–2018	Beheregaray et al. [12]
		South Australia	2011–2012	Hammer et al. [56]
*Nannoperca australis*	Southern pygmy perch	Murray-Darling Basin, Australia	2008, 2011–2018	Beheregaray et al. [12]
		South Australia	2008, 2011–2012	Hammer et al. [56]
*Craterocephalus fluviatilis*	Murray hardyhead	Lodden River system Victoria, Australia	2012–2014	Stoessel [123]
*Mogurnda adspersa*	Southern purple-spotted gudgeon	New South Wales, Australia	2003–2008, 2011	Lintermans et al. [81]
*Galaxias fasciatus*	Banded Kokopu	Kaiwharawhara stream, New Zealand	Prior to 2015	Pham et al. [100]
*Galaxias argenteus*	Giant Kokopu	Nukumea stream, New Zealand	Since 2009	Franklin and Baker [46]
		*Europe*		
*Umbra krameria*	European mudminnow	Szada, Hungary	Since 2008	Bajomi et al. [4]
*Alosa alosa*	Allis shad	Rhine system, Netherlands, Germany, France	Since 2003	Beeck et al. [9]
*Aphanius iberus*	Spanish toothcarp	Valencia, Spain	Since 1992	López and Mata [82]
*Misgurnus fossilis sp.*	European weatherfish	Rhineland-Palantinate and Hesse, Germany	2014–2016	Schreiber et al. [112]
*Cottus rhenanus*	Rhine sculpin	North Rhine-Westphalia, Germany	2015–2020	Hempel et al. [62]
*Chondrostoma nasus*	Nase	River Lahn, Germany	2014–2015	Wetjen et al. [140]
*Acipenser oxyrinchus*	Sturgeon	Gulf of Riga, Latvia	2013–2015	Purvina and Medne [104]
		*Asia*		
*Oncorhynchus masou formosanus*	Formosan landlocked salmon	Shei-Pa National Park, Taiwan	Since 1992	Wu et al. [154]
*Puntius bandula*	Bandula barb	Kegalle district, Sri Lanka	2001	Soorae [120]
*Pseudopungtungia nigra*	Black shinner	Gapcheon, Ungcheoncheon Streams, Korea	Since 2000	Kim et al. [75]

### Drivers of fish reintroduction success and failure

Based on the 260 identified cases of fish reintroductions reported by Cochran-Biederman et al. [24], 42% were classified as failures. Of the further 64 cases identified by the present authors in the existing literature (Table 1), 17% (*n* = 11) were classified as failures, 23% as partial successes (*n* = 15), and 39% as successes (*n* = 25), with 21% (*n* = 13) impossible to classify due to insufficient information available. Similar proportions of failure to Cochran-Biederman et al. [24] were reported in an Australian-based review of fish species translocations, where out of 99 individual translocations since 1980 (covering 17 species), 37% (*n* = 37) were classified as unsuccessful, though exact causes were not specified [81].

In their review, Cochran-Biederman et al. [24] found reintroduction success was mostly attributed to reproductive outcomes: survival (i.e., fish found alive ≥ 6 months post-reintroduction), spawning (i.e., reintroduced fish spawned after reaching sexual maturity), and recruitment (i.e., spawn of reintroduced fish joined breeding population). Following the criteria for partial success classification stated in Cochran-Biederman et al. [24], there were 15 partially successful cases from the studies listed in Table 1. Of those cases, it was insufficient evidence of reproductive outcomes that hindered 8 cases (*n* = 54%) achieving full success. For example, only 4 cases (27%) evidenced survival, but not spawning, recruitment, or long-term self-sustainment, which prevented the individual published studies from classifying their own reintroduction projects as successful. Based on the reproductive outcomes defined by Cochran-Biderman et al. [24], where survival was correlated with project success, these projects could be classified as successes. It should be noted, however, that other literature suggests species reintroduction success must be evidenced by extensive demographic data on spawning, recruitment, turnover, and death [109].

For Cochran-Biederman et al. [24], the definitive causes of reintroduction failure were more varied across the identified studies compared to drivers of success. The variable that most influenced reintroduction outcome was addressing the initial cause of species decline [24]. For example, 65% of failed cases did not address the initial cause of decline, while 68% of successful cases did. The next most important variable determining reintroduction success or failure was habitat quality. Specifically, confirming the presence of required physical habitat was the most important action to avoid spawning failure [24]. While stocking variables were overall less important in determining reintroduction success or failure, the study showed that 71% of recruitment failures were associated with hatchery-reared fish [24]. Though variation linked to intrinsic species characteristics was present (e.g., migratory species more commonly survived for ≥ 6 months after reintroduction than nonmigratory species [94% and 83% respectively]), when investigating the influence of species characteristics further, habitat quality and stocking variables were considered most influential on reintroduction success or failure [24]. Interestingly, of the 11 failures identified from the studies listed in Table 1, issues of habitat quality were only related to 2 cases (18%), while biology and genetics were attributed to failure in 4 cases (36%). Unfortunately, cause of failure could not be determined for the remaining 5 failure cases obtained from the studies in Table 1 due to lack of published information.

### Spatial variation in fish species reintroductions

Of the fish reintroduction cases documented by Cochran-Biederman et al. [24], 75% (*n* = 194) were in North America with most being for riverine fish (60%). Similarly, from the studies listed in Table 1, 45% were North American (*n* = 29) and 38% were from Oceania (*n* = 24), mostly from Australia (*n* = 22). Europe comprised 12% (*n* = 31) of the studies identified by Cochran-Biederman et al. [24] and 11% (*n* = 7) of total cases covered in Table 1.

It is clear that in North America, fish reintroductions are commonly applied conservation tools for conservation and recreational objectives. A review of fish reintroduction projects for protected species in Canada under the Species at Risk Act of 2002 found that reintroduction success relied heavily on having a comprehensive understanding of species ecology and life history, with consideration of genomics being crucial [78]. In addition, as for general species reintroductions, a key part of feasibility assessments will be identifying and ameliorating initial causes of decline. For example, pressures from non-native species are a common cause of population decline for native species and in North America, a common method of non-native fish removal over the last 70 years has been use of piscicides, such as rotenone [43, 142]. Of the North American studies listed in Table 1, 20% (*n* = 6) used a piscicide [30, 48, 77, 114, 124, 156] and a further 24% (*n* = 7) used a mechanical mixed-method approach [20, 27, 36, 63, 110]. Although an important management tool, the use of piscicides, can have undesired secondary impacts on zooplankton and macroinvertebrate communities [8, 28] and in some cases the use of piscicides can fail to eradicate the target species [79]. Stakeholder perceptions are an important additional element to manage when dealing with fish removal methods, particularly piscicides. For example, despite a 95% reduction in non-native sea lamprey *Petromyzon marinus* in the upper Great Lakes, public support for piscicide use was not fully secured with apprehension regarding the risk non-native sea lamprey pose and trust in authorities to make decisions regarding management [47].

In preparing more detailed guidelines for fish reintroductions in the US, George et al. [51] stated that scientifically based protocols for propagation, translocation, reintroduction, and augmentation are the key aspects, which often require diverse skill sets and long-term investment from all parties involved, including collaboration and funding. Examples of fish reintroductions in the US include, lake sturgeon *Acipenser fulvescens* in the 1990s and barrens topminnow *Fundulus julisia* in the 2000s, both in Tennessee*,* with successes in both cases attributed to good public support for the project, increased through public engagement [50]. George et al. [50] state that treating species reintroductions as a long-term investment instead of a temporary project, will allow the public to become invested, which builds trust for subsequent species reintroductions. This is a lesson that most reintroduction projects, including in the UK, can learn from, as some reintroduction programmes have neglected the public [58].

Across Europe, 37% of freshwater fish are threatened and translocations have become an increasingly popular tool [125]. While there is no current review of fish reintroduction projects in Europe specifically, there are several success stories to learn from, as summarised in Table 1. A reintroduction programme in Rhineland-Palatinate and Hesse, Germany for the European weatherfish *Misgurnus fossilis* reported short-term reintroduction success, which reflected the appropriateness of their considerations of stocking strategy and habitat assessments [112]. A reintroduction project for bleak *Alburnoides bipunctatus* in central Germany used environmental DNA (eDNA) monitoring and successfully detected the species at all reintroduction sites, reflecting its potential utility as an inexpensive monitoring tool in the future [106]. In the Carpathian Basin, Hungary, a combination of translocation, captive breeding, restocking, and reintroduction was used to help the endemic European mudminnow *Umbra krameria* under pressure from habitat loss and invasive species [125].

Overall, by analysing existing studies of fish reintroductions, vital lessons can be gleaned to benefit any potential fish reintroduction to the UK. From their in-depth review, Cochran-Biederman et al. [24] showed that inadequate consideration of the cause of initial decline was a common cause of reintroduction failure. This study also found that solving issues associated with habitat quality (i.e., water quality, prey abundance), followed by stocking (i.e., genetic diversity of stocking source, duration of stocking event) were the best indicators of reintroduction outcome. Moreover, studies from the US have shown that reintroductions are not just scientific experiments, but are socio-ecological projects that must involve public and stakeholder engagement to ensure long-term success [50]. In Europe, the essential lessons from recent published studies further suggest that release strategy [125] and employment of appropriate monitoring technologies (e.g., eDNA) are crucial [29, 106].

## UK species reintroductions

There is no doubt that, despite questionable success and limitations, the popularity of species reintroductions globally has increased over time (see Fig. 2.1 in [115]). In the UK, species reintroductions are also increasing in popularity, stemming from the progression of conservation work from intense management and single species focus, to an increasingly landscape-scale, rewilding-based approach [99].

### Policy context

Conservation action in the UK is guided and regulated by international and national legislation and conventions. At an international scale, the multilateral treaty of the Convention on Biological Diversity and its Global Biodiversity Framework for “Living in Harmony with Nature” [132] guides the UK’s national level conservation policy to focus on restoring ecology and halting biodiversity declines. At a UK-level, the goal of halting species decline by 2030 is required by the Environment Act 2021 [143]. While there is no existing legislation specifically for species reintroductions in the UK, they are governed by the Wildlife and Countryside Act 1981, which details requirements for releasing native, non-native, or ‘not ordinarily resident’ species [64]. Aside from statutory obligations, there are vast guidelines relating to species reintroductions provided by both government and NGOs. Hodder and Bullock [64] note 16 guides that were published from 1970 to 1996 covering a range of taxa, including, plants, insects, herpetofauna, and birds. Similarly, they identify 13 international guides ranging from 1976 to 1995. More recently, however, the UK government adapted and published its own guidelines for England in 2021 [31] and continues to publish species-specific guidance as need develops [33].

### General species reintroduction history

A review for the UK found that there had been nine species reintroductions from 1970 to 2016: five birds, one mammal, one amphibian, and two invertebrates (Table 1 in [21]). The earliest, albeit unsuccessful, reintroduction attempt was the release of the large copper butterfly *Lycaena dispar* in 1927 [21]. In 2023, Natural England (a non-departmental public body that advises the UK government on the natural environment) published a UK species reintroduction case studies report, which included projects for the red skipper butterfly *Carterocephalus palaemon*, netted carpet moth *Eustroma reticulatum,* freshwater pearl mussel *Margaritifera margaritifera*, tansy beetle *Chrysolina graminis,* water vole *Arvicola amphibius,* and lesser bladderwort *Utricularia minor* [139].

The 1975 reintroduction for the white-tailed eagle *Haliaeetus albicilla* was the first programme in the UK with the intent of restoring an extinct species for conservation purposes. The mammal reintroduction noted by Carter et al. [21] is the Eurasian beaver *Castor fiber*. The first early trial in enclosures occurred in Scotland, which led eventually to free releases. Other enclosure trials have occurred in the English counties of Norfolk, Devon, and Nottinghamshire, amongst many other locations [105]. Recently, however, free releases have been permitted in England [33]. Since the study of Carter et al. [21], pine marten *Martes martes* have been released in the Forest of Dean, Gloucestershire, western England [92]. There have also been escapes or illegal releases of wild boar *Sus scrofa,* which have begun to establish themselves across England [7], though these should be differentiated from reintroduction projects that follow required policy and legislative steps. Attempts have been made to reintroduce Eurasian lynx [58], grey wolf [87], and brown bear *Ursus arctos arctos* [144], but all have been denied or prevented so far. Public engagement and managing public attitudes are a common obstacle for reintroductions for predators, which is especially true in the UK. For example, the first UK attempts to reintroduce the Eurasian lynx were a failure because insufficient time was invested in researching and gauging public attitudes [58].

The reintroductions conducted in the UK have brought multiple ecological benefits that vary across spatial and temporal scales. For example, the red kite *Milvus milvus* was reintroduced across England and Scotland, beginning in 1989. Following the reintroduction, it was negatively affected by lead ammunition and illegal poisoning, and to prevent this, policy was developed to change ammunition types and better deter illegal poison baiting [93]. This not only protected the red kite population, but benefited other scavenging and predatory species also susceptible to poisoning [118]. Moreover, there are also examples of the umbrella effect in the UK, with habitat restoration work conducted prior to reintroduction benefitting not only the candidate reintroduction species, but also wider biodiversity. For example, the reintroduction of the short-haired bumblebee *Bombus subterraneus* to southeast England required improved management of 850 ha flower rich grassland, which also benefited the rare shrill carder bee *Bombus sylvarum* [49].

It has been estimated that the UK has only 50.3% of its former biodiversity left [66]. Further, landscape fragmentation hinders the mobility of mammals, reptiles, and amphibians [39, 44, 76]. Fragmentation is particularly acute in hydroscapes and has direct impact on fish movement. For example, a recent study found that rivers in Great Britain have on average one barrier (e.g., weir, dam, lock, etc.) per 1.5 km, suggesting that only 1% of rivers in England, Scotland, and Wales are free from artificial barriers [74]. Given the life history traits of many freshwater fish species, such as migration, aquatic fragmentation can be detrimental to fish populations. In the UK, habitat modification impacts on freshwater species is compounded by current (and future anticipated) effects of climate change. With air temperature set to increase across the UK and precipitation patterns less certain, species reintroductions in the form of ‘assisted colonisation’ might become increasingly popular in UK conservation in the future [21].

Overall, the UK has utilised species reintroductions as a conservation tool for the benefit of biodiversity for multiple taxa previously, but at the time of writing (i.e., 2025), no reintroduction of an extirpated fish species has been attempted. By utilising the experiences of extensive global fish reintroduction case studies, the UK could be poised to change this position soon for its extirpated fish species – the burbot.

## Considering a UK fish species reintroduction

The UK has 55 species of freshwater fish, 13 of which have been introduced by humans [83, 91]. There have been known extinctions of burbot and sturgeon *Acipenser sturio* across the UK, with some local extinctions of whitefish populations (i.e., vendace *Coregonus albula* and *Coregonus vandesius*) in Scottish lakes [147]. Moreover, salmon *Salmo salar* and European eel *Anguilla anguilla* populations are currently declining [18], thus it can be concluded that freshwater fish populations are pressured in the UK. Given this trend combined with and the popularity of species reintroductions for other taxa, it is likely that fish reintroductions could become a common conservation tool in the future [21].

Based on this global review of multi-taxa reintroductions, the key considerations for a fish species reintroduction in the UK would be: addressing initial causes of decline [24]; providing sufficient quality release habitat [13, 24]; designing an appropriate release strategy that considers genetics as well logistics [24, 78, 112, 125]; establishing a comprehensive understanding of the specie’s biology as well as the science behind all stages of the reintroduction process [13, 51]; securing sufficient funding and applying appropriate post-release monitoring [13, 15, 29, 106], and appropriate engagement with social and political stakeholders [50]. In a UK context, these challenges can be assessed against a proposed burbot reintroduction.

### A potential future English fish reintroduction – Burbot

#### Burbot background

**Fig. 1:**
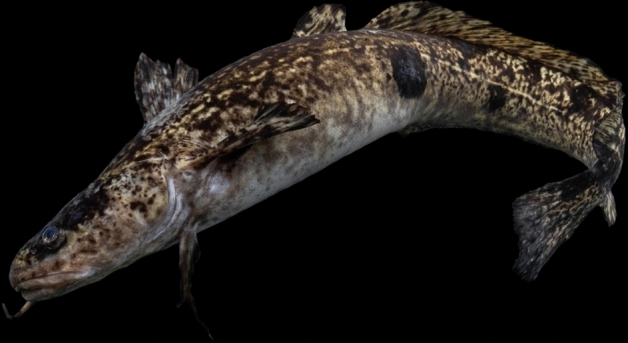
Burbot (*Lota lota*) photographed at gavins point national fish hatchery in Yankton, South Dakota, US by Sam Stukel (USFWS), 2023

**Fig. 2:**
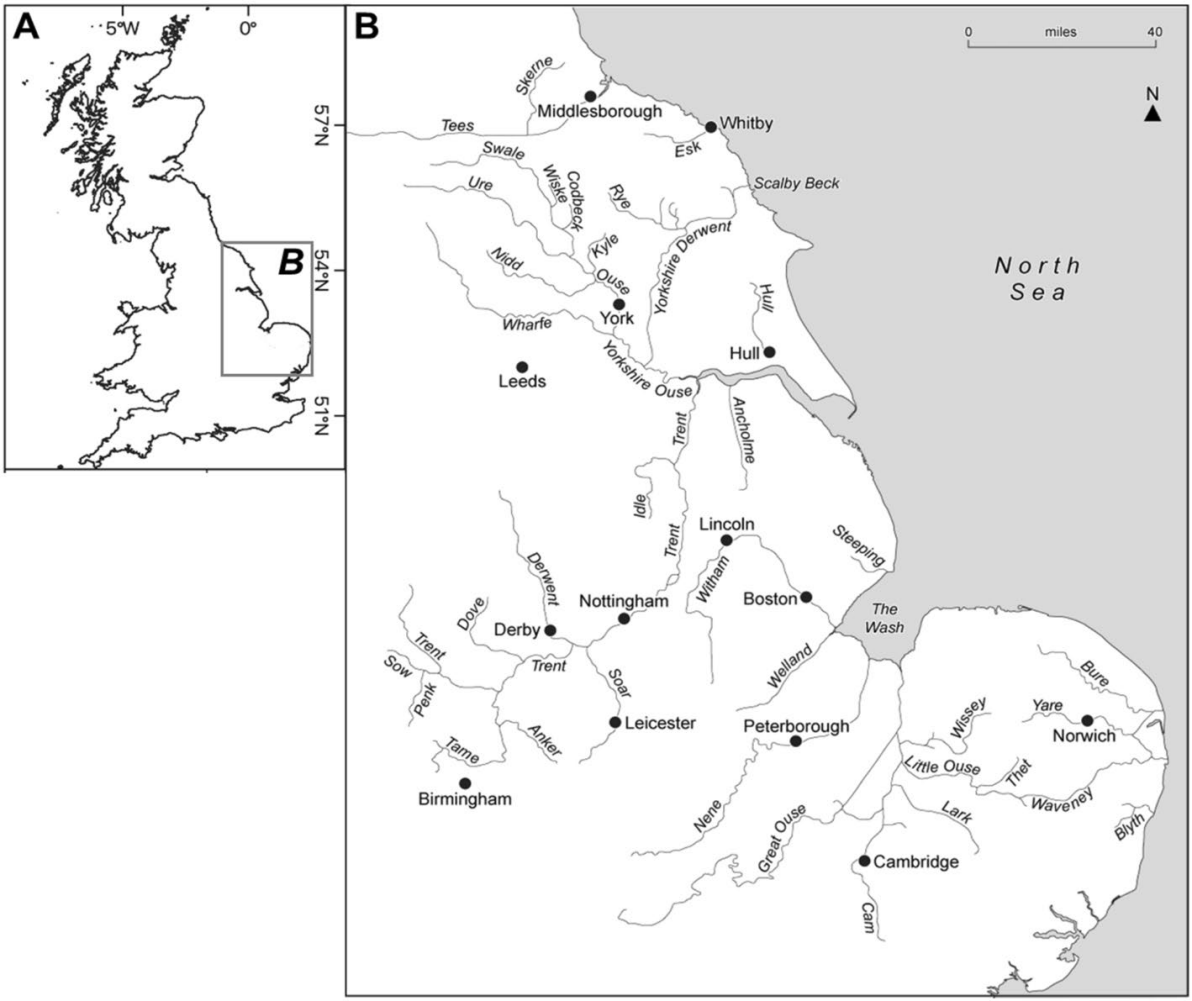
Historical distribution of the burbot *Lota lota* in England. **A** Great Britain indicating location of **B** burbot rivers of England. Adapted with permission from Worthington [151]. Original figure published in Pearce et al. [97]

The burbot (Fig. 1) is the only freshwater member of the order Gadidae and has a circumpolar distribution [121, 149]. Adult burbot have been recorded to reach 120 cm length and 30 kg weight in Siberia and Alaska, but in England (prior to extirpation) they ranged from 30 to 60 cm in length [148]. 42 eastern flowing rivers in England have been identified as previously supporting burbot as indicated by historical records (see Fig. 2, [97, 151]). Burbot were extirpated in the 1960s, with the last recorded catch in the Old West River, near Aldreth, Cambridgeshire, in 1969. Its extirpation has been linked to widespread river channelisation and river-floodplain disconnection that reduced the availability of spawning and larval habitats [149]. The burbot’s lifecycle, especially its floodplain phase, represents a challenge to a successful reintroduction as historical modification of rivers has resulted in a reduction in connectivity to lateral habitats in England.

#### Attempting an English burbot reintroduction

As emphasised by the literature, species reintroductions are not short-term fixes, but require long-term, well-designed management plans that address the many potential pitfalls that can lead to failure. To adequately address these considerations, for the burbot reintroduction in England, practitioners could apply a management framework to ensure conservation practice is effective. To exemplify this, future considerations for the burbot reintroduction to England are discussed in the context of the *Open Standards for the Practice of Conservation* (hereafter CS [25])*.* This decision framework mirrors the planning and implementation stages found in the IUCN’s *Guidelines for Reintroductions and Other Conservation Translocations* [70]. These guidelines have several distinct stages: planning, feasibility, risk assessment, release strategy, monitoring, long-term management, and dissemination. For the CS, the stages are: ‘assess’, ‘plan’, ‘implement’, ‘analyse and adapt’, and ‘share’.

In the first ‘assess’ stage of the CS, it is fundamental to define project purpose and identify a project team, define the scope, vision, and conservation targets, and identify critical threats [25]. For the English burbot reintroduction, it is crucial that the project team be comprised of all relevant stakeholders: NGOs, statutory authorities, anglers, landowners and potentially others. The scope of the project itself must consider both spatial and temporal dimensions. For example, the overall project goal could be to ‘restore a self-sustaining population to the historical range of burbot in England’, from which the spatial scope would cover eastern flowing rivers in England (see Fig. 2). As reintroduction projects increase in scale spatially, the project is also increasingly dependent on effective coordination and communication with multiple stakeholders [54]. For example, the conservation of the bull trout *Salvelinus confluentus* in the US has been ongoing since 1999 and has involved a variety of local and regional partnerships and collaborations between state-level fish and game authorities, state and federal land management and water resource agencies, tribal governments, utility companies and private groups, water users, ranchers, and landowners [127]. The project, which expanded across six states [59], was organised into six recovery units and broken down into 109 core areas to organise tasks and teams [127]. Collaboration and project management was organised using decision support tools, such as structured decision-making [127], which has resulted in localised project success evidenced by restored and naturally reproducing bull trout populations [141].

The reintroduction projects discussed in this review, as well as the CS, especially emphasises the importance of identifying threats to the success of a reintroduction project, including ameliorating initial causes of species decline, insufficient understanding of species biology, and inadequate release habitat [13, 24]. For example, Castañeda et al. [22] found the eradication of an invasive species, smallmouth bass *Micropterus dolomieu* in the Rondegat River in South Africa, allowed for the recolonisation and recovery of several endemic native fish species, such as Clanwilliam redfin *Sedercypris calidus* over a 10-year period. This example emphasises the importance of ameliorating the initial cause of decline before attempting a species reintroduction as by eradicating, in this case, the invasive species, the fish population recovered on its own making a species reintroduction unnecessary. Therefore, in any situation, before undertaking the steps of the translocation guidelines, the appropriateness of a species reintroduction to achieve conservation goals must be evaluated because it is not the panacea of all biodiversity declines.

In terms of the English burbot reintroduction, overcoming barriers to reintroduction implementation necessitates good supporting science, which for the burbot reintroduction has been advancing since the 2000s. Research into the feasibility of an English burbot reintroduction has specifically addressed the pre-release knowledge requirements as stated in the IUCN translocation guidelines [70]. Past studies have identified reduction in lateral floodplain spawning habitat as the cause of initial decline [148, 149] and sites of sufficient quality and quantity have been identified [95, 96, 150]. As highlighted by Cochran-Biederman et al. [24], insufficient research regarding genetics can directly hinder fish reintroduction success. While the closest genetic relative of English burbot has been identified in previous research as the western European subclade [131, 153], more planning is required related to stocking strategy. With stocking strategy for example, there are multiple approaches. For burbot reintroductions in western Europe, it has been shown that annual releases are required for a minimum of 10 years to obtain first evidence of natural reproduction [11, 137], but the age-group to be stocked varies. In the current Belgian reintroduction project, larvae are released annually to the Grote Nete system, Antwerp, Belgium [34, 134], as was similar to the stocking strategy in the Beerze River, Netherlands from 2009 to 2013 [11]. In the North Rhine-Westphalia region, Germany, however, they annually stock fingerlings (Scharbert, A., pers. comms., 2025). Each has advantages: stocking larvae means that more individuals can be released while reducing rearing costs, but by stocking fingerlings the chance of individual survival is increased as they have greater swimming capacity.

Another essential element of the ‘assess' stage is the identification of all stakeholders and the determination of their primary interests [25]. For example, stakeholder engagement is crucial, as demonstrated by previous reintroduction attempts in the UK [58]. The burbot is a top-level predator, which can co-exist with other predatory species [122], but has an ecological role in English rivers. While no ecosystem engineer, the burbot’s requirements for different river-floodplain habitat niches across its life cycle would mean active restoration of lateral and longitudinal river-floodplain habitat. The benefits this species could bring to the environment should be clearly communicated to the public to assuage fears connected to predators. Key stakeholders to engage with, for example, would be local anglers. While this was done previously in the early 2010s at a national-level and found > 90% of respondents supported the reintroduction [152], it is important this work is repeated as opinions might have changed. Throughout much of its natural range the burbot is an important fishery species (e.g., North America, Scandinavia, Russia, and China) [42], thus it could bring recreational value to English rivers and may receive support even from anglers who target large bodied predatory fish species [45, 53, 89]. Though, this support cannot be assumed and investment into public engagement must be undertaken. Thus, it is recommended that for the English burbot reintroduction, an assessment of local stakeholder attitudes at the candidate reintroduction site should be undertaken. In the case of the burbot reintroduction, it would also be important to consider the interests of and engage with waterfront property and land owners and the authorities that manage flood control infrastructure because of the importance of floodplain habitat for spawning. It would be crucial that the project identifies key geographical working areas and that it engages with all affected persons throughout the project. Importantly, the floodplain restoration work that could be necessary to the project is currently synchronous with national level policy, such as Natural Flood Management within the National Flood and Coastal Risk Management Strategy for England [41] and Environmental Land Management [32].

Multiple aspects of a reintroduction project require extensive planning. In the second ‘plan’ stage of the CS, individual action, monitoring, and operational plans are required, which is supported by a work plan that is a short-term schedule of implementation [25]. Combined, these formulate an overall strategic action plan. Individual action plans need to set goals for each conservation and human well-being target in the project, and should include strategies for key intervention points, and objectives for key immediate results. Monitoring plans highlight indicators of project success and methods to evaluate them, identification of the data recipients and the preferred communication modes, as well as assessments of timescales. Post-release monitoring has been widely emphasised as being critically important to species reintroduction projects [13]. Examples of monitoring plans can be drawn from the Netherlands and Belgian burbot reintroductions, which have used mixed methods to monitor different burbot life stages annually [94, 128, 129, 133, 137]. In Belgium, for example, electrofishing is used to monitor adult and juvenile abundance and distribution, while light traps and visual surveys are used to survey larvae. Burbot releases have occurred in 15 rivers since 2005 [130] with the Grote Nete having the most consistent annual releases. Over this period burbot have been found in an additional 10 rivers. Combined with evidence of natural reproduction, could this project be classified as successful? It is crucial that all monitoring plans consider which success indicators are most appropriate for the project and the cost-effectiveness of the methods used to monitor them. For example, the Netherlands and Belgium reintroductions have demonstrated the difficulty in documenting survival rates and evidencing natural reproduction due to the difficulty in locating spawning sites. It is thus crucial to apply a mixed methodology (i.e. electrofishing, bioacoustics, eDNA, and visual surveys) to identify all life stages, which cumulatively, make monitoring resource intensive. These biological indicators are one component of a reintroduction project, and thus, indirect indicators such as socio-economic factors such as support or attitudes during the reintroduction, should be considered. Finally, operational plans relate to funding, human capacity, skills, risk assessment and mitigation, as well as exit strategy.

The ‘implementation’ stage that follows is crucial. Based on the prior development of the strategic plan, a reintroduction project needs to be further compartmentalised to create detailed, short-term work plans to achieve conservation targets set out in the action plans. These work plans require refined budgets, funding applications and acquisition, and delegation of project tasks to staff and work units, combined with implementation of monitoring, data storage, analysis, and dissemination [25].

Following implementation, the next stage is to analyse all data collected (i.e., both scientific and financial) and adapt the project strategy based on key findings [25]. It is crucial to understand and plan how the data collected is going to be analysed and how the outputs of the project will be used. It is common in conservation projects that data can go unanalysed or underutilised due to issues with timing during project implementation [25]. In terms of the burbot project, if a multi-method monitoring methodology is applied to surveying (e.g. electrofishing, eDNA, and hydrological acoustics), the data analysis will be resource intensive requiring expertise from multiple fields: genetic biology, bioacoustics, and fisheries science. For the burbot reintroduction in the UK, the slow recovery time and approximate 10-year period to evidence natural recruitment observed in both Belgium and the Netherlands [10, 11, 137] suggests any burbot reintroduction programme in England will require long-term investment and monitoring. The Belgian and Dutch reintroduction projects have applied mixed-method approaches to monitoring and evaluation and have utilised collected data to review and adapt their approaches. This adaptive management is crucial to reintroductions and conservation projects more generally, as monitoring alone cannot increase evidence-based decision making as data outputs must be translated into effective conservation action [80].

The final ‘share’ stage in the CS guidance is related to disseminating findings [25]. As is clear from the guidance, as well as the current review, documenting lessons learned and disseminating information is crucial to existing and future species reintroduction projects. The English burbot project will require ongoing collaboration of multiple stakeholders and the communication of findings will require multiple outputs: media engagement, scientific papers, reports, and presentations for experts and non-experts. Moreover, sharing findings via open conservation databases such as Conservation Evidence, Panorama, or MiradiShare would help to mitigate some common pitfalls in future projects by increasing knowledge exchange and the spatial impact of the project’s findings [84].

## Conclusions

Species reintroductions, and specifically fish reintroductions, have had varied success globally. While spatially biased towards North America, there are lessons to be learned from all cases that can benefit any future reintroduction attempt. In particular, when considering a UK fish species reintroduction: causes of original decline must be addressed and there must be adequate release habitat. Further, understanding the biology of the species to be reintroduced is paramount, with this dictating the requirements of pre-reintroduction work aimed at identifying and restoring appropriate habitat for release. Using the case study of a burbot reintroduction to England, previous research has addressed some key concerns, but it is unclear what barriers remain in the scientific evidence basis as well as compliance with existing policy. For the former, the development of a release strategy, as well as the implementation of pre- and post-release monitoring of existing fish and wider ecology is urgently needed. All these considerations would be incorporated if an adaptive management framework, such as the *Open Standards for the Practice of Conservation,* is applied. Not only would this ensure the health of a reintroduced burbot population is monitored, but further allows any potential lessons learned to be reported and such an approach would then contribute to existing literature to help guide the application of species reintroductions as a tool within conservation science.

## Data Availability

The data that support the findings of this study are available from the corresponding author, RHP, upon reasonable request.
